# Global research trends and hotspots on glioma stem cells

**DOI:** 10.3389/fonc.2022.926025

**Published:** 2022-09-29

**Authors:** Sirong Song, Haiyang Wu, Fanchen Wang, Jiji Jiao, Lixia Xu, Hongguang Wang, Xiaoguang Tong, Hua Yan

**Affiliations:** ^1^ Clinical College of Neurology, Neurosurgery and Neurorehabilitation, Tianjin Medical University, Tianjin, China; ^2^ Tianjin Neurosurgical Institute, Tianjin Key Laboratory of Cerebrovascular and Neurodegenerative Diseases, Tianjin Huanhu Hospital, Tianjin, China; ^3^ Department of Neurology, Tianjin Huanhu Hospital, Tianjin, China

**Keywords:** glioma stem cell (GSC), chemotherapy resistance, EMT - epithelial to mesenchymal transformation, hotpots, bibliometric analysis

## Abstract

**Background:**

Glioma stem cells (GSCs) are a sub-population of cancer stem cells with capacity of self-renewal and differentiation. Accumulated evidence has revealed that GSCs were shown to contribute to gliomagenesis, distant metastasis as well as the resistance to radiotherapy and chemotherapy. As a result, GSCs were regarded as a promising therapeutic target in human glioma. The purpose of our study is to identify current state and hotspots of GSCs research by analyzing scientific publications through bibliometric methods.

**Methods:**

All relevant publications on GSCs during 2003-2021 were extracted from the Science Citation Index Expanded of Web of Science Core Collection (WoSCC), and related information was collected and analyzed using Microsoft Excel 2016, GraphPad Prism 8 and VOSviewer software.

**Results:**

A total of 4990 papers were included. The United States accounted for the largest number of publications (1852), the second average citations per item (ACI) value (67.54) as well as the highest H-index (157). *Cancer Research* was the most influential journal in this field. The most contributive institution was League of European Research Universities. RICH JN was the author with the most publications (109) and the highest H-index (59). All studies were clustered into 3 groups: “glioma stem cell properties”, “cell biological properties” and “oncology therapy”. The keywords “identification”, “CD133” and “side population” appeared earlier with the smaller average appearing years (AAY), and the keywords”radiotherapy” and “chemotherapy” had the latest AAY. The analysis of top cited articles showed that “temozolomide”, “epithelial-mesenchymal transition”, and “immunotherapy” emerged as new focused issues.

**Conclusion:**

There has been a growing number of researches on GSCs. The United States has always been a leading player in this domain. In general, the research focus has gradually shifted from basic cellular biology to the solutions of clinical concerns. “Temozolomide resistance”, “epithelial-mesenchymal transition”, and “immunotherapy” should be given more attention in the future.

## Background

Glioma is one of the most common primary malignant tumors of the central nervous system, accounting for 45%-60% of adult intracranial tumors, with a 5-year survival rate of less than 10% (1, 2). Glioma stem cells (GSCs) are a particular group of cells within the glioma mass, which also were known as glioma-initiating cells (3, 4). Previous reports have demonstrated that GSCs share fundamental stem cell properties of self-proliferation and multi-lineage differentiation. Emerging evidence suggests that GSCs to a large extent contribute to glioma recurrence and therapy resistance (5, 6). For example, radiotherapy is particularly effective against rapidly proliferating tumors cells, while GSCs are mainly quiescent in the G0 state, and therefore allows them to exhibit high resistance to radiotherapy. In addition, GSCs are able to expel antineoplastic drugs to the extracellular medium by using multidrug resistance-associated protein transporter (7, 8). In view of this, GSCs are considered to be critical therapeutic targets for glioma relapse and drug resistance (9).

Appropriate epistasis regulation is vital for the maintenance of GSCs. Temozolomide (TMZ) is the first-line chemotherapeutic agent for glioma, and main mechanism of action of this drug is to promote methylation of guanine in DNA, leading to glioma cell cycle arrest (10). In contrast, O-6-Methylguanine-DNA methyltransferase (MGMT) is a key enzyme for DNA repair (9, 11), which functions as removing alkyl groups from guanine residues, thereby counteracting TMZ-induced DNA damage, an important mechanism in the resistance of glioma cells to TMZ treatment. Multiple studies have shown that miRNA and LncRNA could reduce MGMT activity levels to improve TMZ sensitivity (12, 13). And they could regulate the growth pathway of GSCs, promote apoptosis and inhibit cell proliferation. Additionally, some miRNAs were confirmed to be closely associated with glioma prognosis and can also be used as prognostic predictors (14).

Meanwhile, GSCs have been isolated mainly from glioma cell lines cultured *in vitro* or human brain tumors. Most GSCs’ markers are derived from normal stem cells, including SOX2, NANOG, OLOG 2, MYC, BMI1 and differentiation inhibitor protein 1 (ID1), as well as surface markers CD133, CD24, CD44 and Nestin (3, 15). These markers can be used as an indicator for GSCs identification and also for cell sorting (16). However, GSCs cultured *in vitro* may not accurately reflect their physiological status as they showed *in vivo*, even though the glioma cells were derived from patients. As GSCs *in vivo* are exposed to the presence of large numbers of immune cells, complex regulation of substance metabolism and growth regulation, it is difficult to recreate the real context, even with three-dimensional (3D) cell cultures and organ models (17, 18).

Given the importance of GSCs in tumor progression and therapeutic resistance, an increasing amount of GSCs research has been carried out in recent years. However, few attempts have been undertaken to analyze the quantity and quality of publications in this field from a global perspective. Most of the studies focus only on one or some particular areas of GSCs research. Therefore, it is necessary to adopt appropriate statistical methods to reveal the overall knowledge framework, research hotspots, and future directions of this field.

Bibliometrics is a scientific discipline employing mathematical, statistical and other econometric methods to explore the nature of scientific activities (19, 20). This method could quantitatively reveal the development history, research focus and future research direction of the scientific field. Bibliometric analysis is now widely used in various fields, including mathematics (21), physics (22), forestry (23), agronomy (24) and medicine (25). However, to the best of our knowledge, no studies involving the bibliometric analysis of GSCs had been conducted. Thus, in the present study, we performed a bibliometric analysis through collecting publications related to GSCs research, and further using VOS viewer software, a widely used bibliometric software to estimate the contribution of countries/institutions/authors, and to identify research trends and hotspots in this field.

## Materials and methods

### Data sources and search strategies

**Figure 1:**
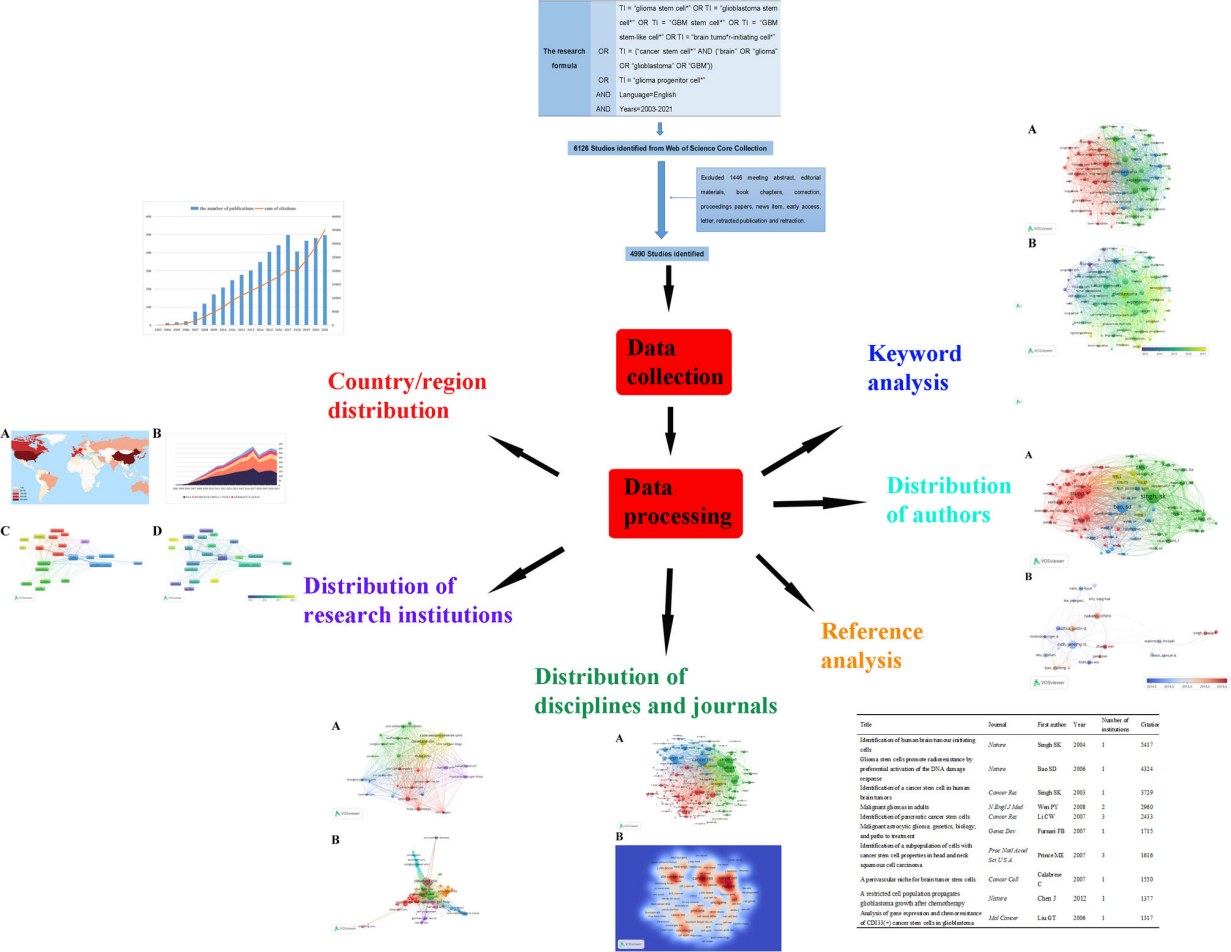
We searched the Web of Science Core Collection (WoSCC) on GSCs and performed a visualization analysis from the perspectives of countries, institutions, disciplines, journals, authors, references and keywords to predict future research hotspots in this field.

Web of Science (WoS) is one of the most authoritative and well-known citation databases, currently covering more than 18000 journals and 256 subject categories. To date, there has been general agreement that the Science Citation Index-Expanded (SCI-EXPANDED) of Thomson Reuters’ WoS Core Collection (WoSCC) is the most appropriate database for conducting bibliometric analysis (19, 20, 25). In this bibliometric research, data were retrieved and downloaded from SCI-EXPANDED (1998-present) of WoSCC. All the search works were completed within the same day in order to avoid the possible bias caused by the daily updating on WoSCC database. Since all data in this study were obtained from public databases and did not require any interactions with human or animal subjects, ethical consent was not applicable. We performed a visualization analysis on the number of publications, citations, and research trends by country, author, and institution using VOSviewer and other software to predict future research hotspots in this field (**Figure 1**).

**Figure 2:**
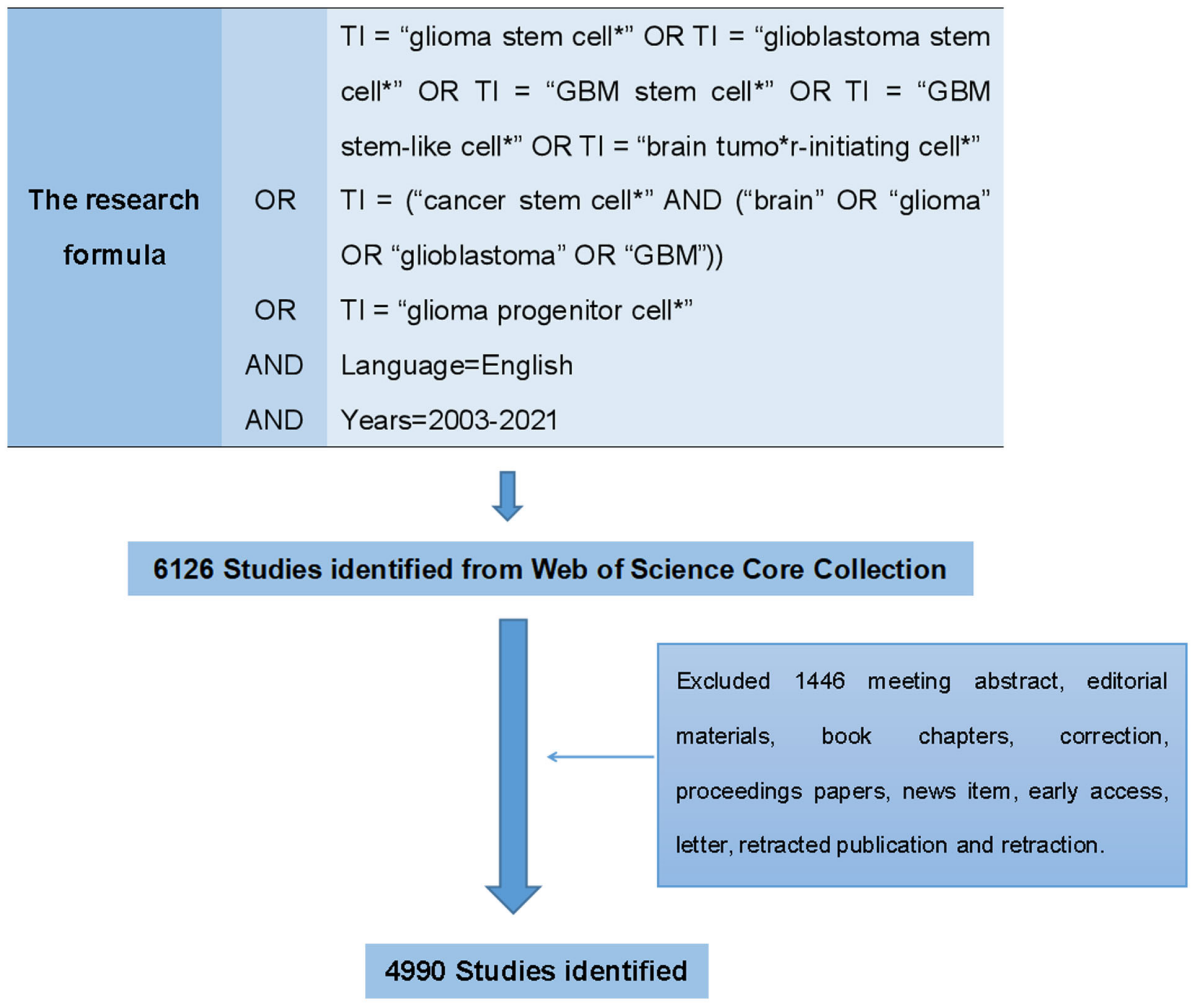
The flowchart of data collection about GSCs on WoSCC.

The search formula was set to TI = “glioma stem cell*” OR TI = “glioblastoma stem cell*” OR TI = “GBM stem cell*” OR TI = “GBM stem-like cell*” OR TI = “brain tumor-initiating cell*” OR TI = (“cancer stem cell*” AND (“brain” OR “glioma” OR “glioblastoma” OR “GBM”)) OR TI = “glioma progenitor cell*” AND language=English. The retrieval time span was limited to 2003-2021 (**Figure 2**). There were 3905 original articles, which accounted for 63.75% of the total number of records, making articles the most frequent document type. Review articles ranked second with 1085 records, comprised 17.71% of the total.

### Data collection

To optimize data analysis, only original articles and reviews were included in the final bibliometric analysis based on previous studies. All retrieved records were assessed and reviewed independently by two investigators (SSR, WHY). The exported data included the number of publications, citation frequency, active countries/regions and institutions, influential authors and journals, disciplines, H-index, average citations per item (ACI) and sum of the times cited (SOTC). Then the data were inputted into Microsoft Excel 2016 and GraphPad Prism 8 and analysed both quantitatively and qualitatively by descriptive analysis.

### Bibliometric analysis

Microsoft Excel 2016 was applied to generate a prediction model: f(x) = ((number of publications in the last year ÷ number of publications in the first year)1/(last year - first year)- 1) × 100, in which we analyzed the time trend of the publications as well as the future change tendency based on the cumulative number of publications (20). The index of H means that a scholar/country has published H papers and that each of them has been cited at least H times by other publications. It is widely accepted that H-index could quantify both the scientific impact and research output of a scholar or a country (21, 22). In addition, a citation is a sign that one’s research has recognized by other researchers and also a way for other scholars to draw on or directly prove their point. Usually speaking, the higher the number of citations, the more attention the result has received and the higher its academic value. Therefore, SOTC and AIC are the frequently used indicators to assess the scientific performance and contribution a scholar or a country (25, 26).

Bibliometric analysis, which takes advantage of mathematical and statistical approaches, is a comprehensive analytical method first defined by Pritchard in 1969. It is regards as one of the optimal methods for quantifying the content of literature and analyzing the correlation of highly cited references with productive authors. The java program VOS viewer (version 1.6.16, Leiden University, The Netherlands, downloadable at www.Vosviewer.com) is one of the most popular bibliometric software tools used for constructing and visualizing the network of authors, country, institution, journal, and keywords. VOS viewer could provide three types of visualizations map including the network visualization map, the overlay visualization map, and the density visualization map (27). Take network visualization map as an example, items are represented by their label and the size of the label is determined by the weight of the item. The links between items indicate the correlation between parameters and the thickness of the link represents the strength of the link. Total Link Strength (TLS) which means the weighed links of the selected nodes is often used to quantitatively evaluate the total links (25, 26). In addition, VOS viewer could classify countries, journals, institutions, and keywords into different clusters based on co-authorship, citation, co-citation, as well as co-occurrence analysis. A detailed explanation for these maps is available in a handbook which could be downloaded from the following Web site: https://www.vosviewer.com/documentation/Manual_VOSviewer_1.6.16.pdf.

## Result

### Temporal distribution map of the scientific literature

**Figure 3:**
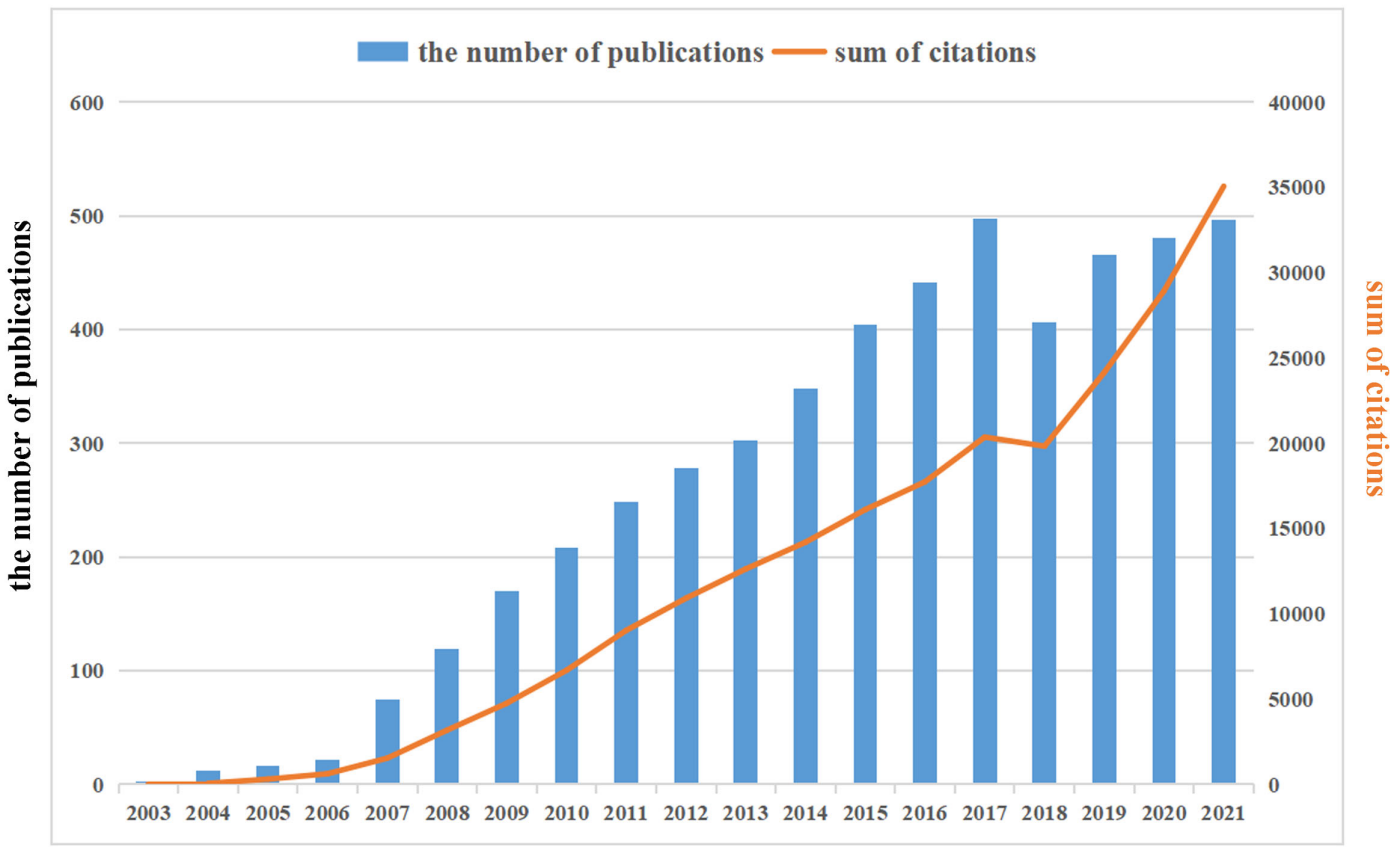
The annual publications and citations annual number of publications and citations in the field of glioma stem cells (GSCs) from 2003 to 2021. There is an increasing trend in GSCs.

Already in 2003, *Cancer Research* published an article by Canadian scholar Singh et al, entitled “Identification of a cancer stem cell in human brain tumors”, which defined a class of brain tumor stem cell for the first time. Starting in 2007, the annual volume of publications related to GSCs has increased steadily, reaching a peak in 2017 (498), accounting for 9.98% of total publications, subsequently with a minor fluctuation in the literature published in 2018 (406) and a rebound in 2019. The annual growth rate from 2007 to 2021 was 14.56%, representing 98.98% of all papers meeting the criteria (4939/4990). In addition, as also can be seen from **Figure 3**, the annual number of citations showed an overall increasing trend, which was similar with the growth pattern of annual number of publications.

### Country/region distribution

**Figure 4:**
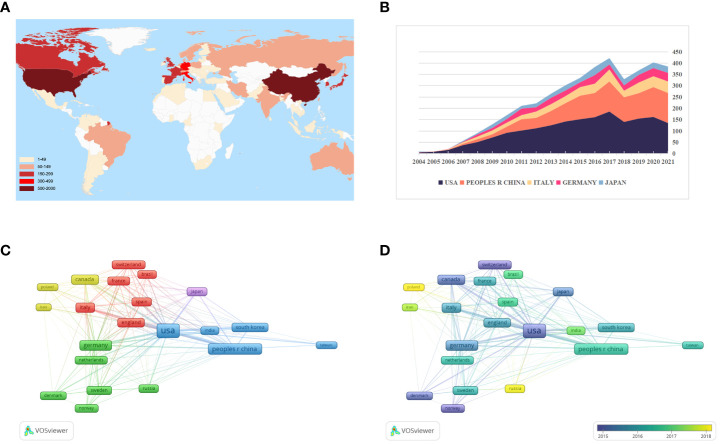
**(A)** The global distribution of different countries involved in glioma stem cells (GSCs) research based on their number of publications. The United State pays the most attention to GSCs, followed by China and Italy. **(B)** The growth trends of annual number of publications in the top 5 prolific countries during the period 2003-2021. The network visualization map **(C)** and overlay visualization map **(D)** of country co-authorship analysis generated by VOS viewer. Countries with the minimum number of 50 publications were assigned into five clusters.

Of these 4990 articles and reviews, 85 countries are involved in GSCs research. From **Figure 4A** and **Table 1**, it can be seen that the United States was the most prolific country with 1852 (37.11%) publications, followed by China (1133, 22.71%) and Italy (469, 9.40%). In terms of the H-index, the United States also ranked first. **Figure 4B** presents the number of papers published per year in the top 5 countries during the period 2003-2021. There has been a consistent growth in annual number of studies on GSCs in the United States since 2004, and culminated in 2017 (186). China has made great progress in this field since the 2010s, but the number of research publications was still lower than the United States.

**Table 1:** The top 10 countries in research scope of glioma stem cells.

Country	Quantity	% of 4990	H-index	ACI	TLS
USA	1852	37.114	157	67.54	739
Peoples R China	1133	22.705	81	31.33	301
Italy	469	9.399	68	43.84	184
Germany	355	7.114	66	52.19	201
Japan	299	5.992	60	40.51	89
South Korea	289	5.792	48	30.39	93
Canada	281	5.631	62	85.98	147
France	216	4.329	45	30.08	84
England	201	4.028	51	42.52	114
Spain	157	3.146	32	29.52	65

ACI, average citations per item.

TLS, Total link strength.

**Figure 4C** illustrates the country co-authorship network visualization map. A total of 22 countries with the minimum number of 50 publications were selected. Each colored node represents a country, and nodes with the same color are grouped into the same cluster. Particularly, the thicker the line between two nodes indicates the closer cooperation between countries. As evident from **Figure 4C**, global country cooperation is broadly divided into five main clusters. The United States has a central position in collaboration with other countries, and collaborated most closely with China, Italy, and England. **Figure 4D** shows the country co-authorship overlay visualization map. The color of each node indicates the average appearing year (AAY) of each country according to the color gradient presented in the lower right corner. From this figure, we can find that the countries of Russia, Poland and Iran were the relatively new entrants in this field. And as one of the early pioneers to focus on GSCs, although Canada is ranked only seventh in terms of total publications, its average number of citations is the highest (85.98).

### Distribution of research institutions

**Figure 5:**
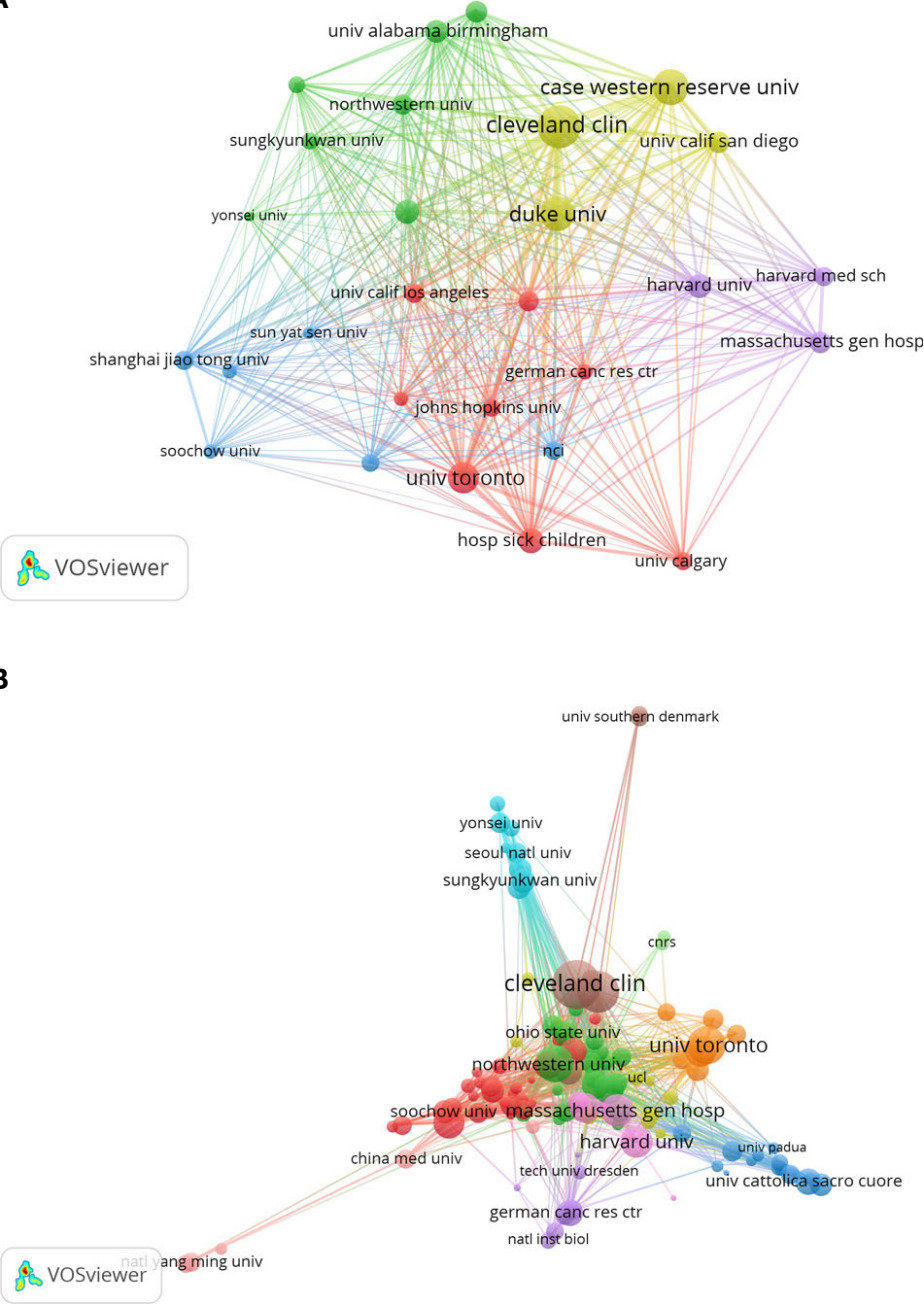
Institution citation map **(A)** and co-authorship network map **(B)** in regard to the research on glioma stem cells research. The connection between the nodes represents a co-citation relationship. Thickness of the line reflects the frequency of the cooperation. And the thicker the line, the stronger the cooperation.

The top 10 institutions in terms of publication on GSCs research were shown in **Table 2**. Of these, there were 7 American institutions, 2 French institutions, and 1 European institution. The League of European Research Universities holds the largest number of publications (289, 5.792%), citations (17623), and the highest value of H-index (66). The citation analysis between institutions is illustrated in **Figure 5A**, the size of the node indicates the centrality, that is TLS. From this figure, it can be seen that Cleveland Medical Center, Case Western Reserve University, Duke university, and University of Toronto occupied the center location of citation. While in terms of ACI, University of Toronto ranks first with 126.38 times, followed by Cleveland Medical Center (84.48) and Harvard University (80.98). Additionally, an institution co-authorship network map with 120 nodes and 1196 links was also generated (**Figure 5B**). Institutions with more than 20 documents were analyzed and the top 3 institutions with the largest TLS were Cleveland Medical Center (134 times), Case Western Reserve University (101) and University of Toronto (88).

**Table 2:** The top 10 research institutions in regard to the research on glioma stem cells.

Institute	Quantity	% of 4990	Country	H-index	ACI	SOTC
League of European Research Universities	289	5.792	European	66	60.98	17623
University of California system	205	4.108	USA	58	67.03	13741
University of Texas system	198	3.968	USA	55	70.9	14038
Harvard University	193	3.868	USA	55	80.98	15629
Cleveland Clinic Foundation	168	3.367	USA	65	84.46	14189
Institut National de la Sante et de la Recherche Medicale	148	2.966	France	40	29.22	4325
UTMD Anderson Cancer Center	140	2.806	USA	49	59.64	8350
University of Toronto	136	2.725	USA	46	126.38	17188
Case Western reserve university	124	2.485	USA	49	66.4	8234
Centre national de la recherche scientifique cnrs	112	2.244	France	31	22.64	2536

ACI, average citations per item.

SOTC, sum of the times cited.

### Distribution of disciplines and journals

Of the 4990 included documents, 18 subject categories were identified. **Table 3** lists the top 20 disciplines of GSCs based on the number of publications. It can be seen that the subject categories receiving the most interest were oncology (42.645%), cell biology (22.926%), biochemistry molecular biology (13.607%) and medicine research experimental (8.236%). Also, the literature in this field was also within the subject areas of pharmacology, genetic inheritance, materials science, and immunology.

**Table 3:** The top 20 disciplines on glioma stem cells.

Disciplines	Quantity	% of 4990
Oncology	2128	42.645
Cell Biology	1144	22.926
Biochemistry Molecular Biology	679	13.607
Medicine Research Experimental	411	8.236
Clinical Neurology	394	7.896
Neurosciences	360	7.214
Multidisciplinary Sciences	347	6.954
Pharmacology Pharmacy	319	6.393
Cell Tissue Engineering	227	4.549
Genetics Heredity	209	4.188
Biotechnology Applied Microbiology	199	3.988
Pathology	172	3.447
Chemistry Multidisciplinary	171	3.427
Hematology	115	2.305
Biophysics	106	2.124
Chemistry Medicinal	102	2.044
Surgery	102	2.044
Immunology	97	1.944
Nanoscience Nanotechnology	72	1.443
Materials Science Biomaterials	59	1.182

**Figure 6:**
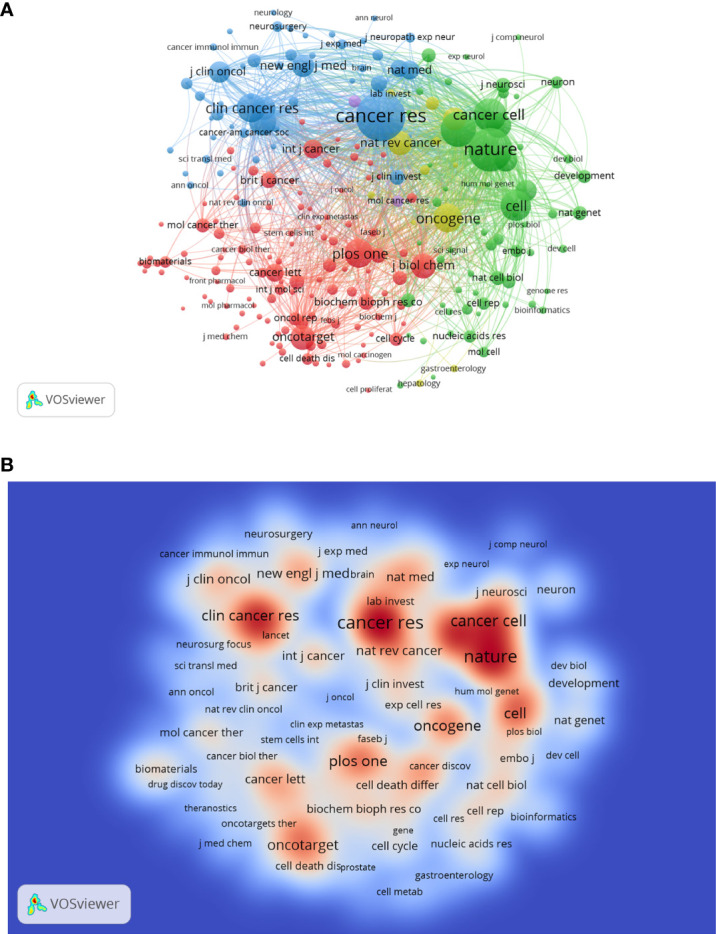
The network visualization map **(A)** and density visualization map **(B)** of journal co-citation analysis. In the visualization map, each node represents a journal and its size is proportional to the number of publications. The deeper the color of a node, the more frequently of keywords appears.

A total of 921 journals have published related articles in this field. **Table 4** shows the distribution of the top 10 productive journals in this area. *Oncotarget*, *Plos One* and *Cancers* are the top 3 contributors with 202, 158 and 132 papers, respectively. *Cancer Research* has the largest H-index of 61, followed by *Stem Cells* (45) and *Oncotarget* (45). As for ACI, *Cancer Research* is the top-ranked with 148.62 times, much higher than other journals. A co-citation analysis was also performed to evaluate the connection among different journals. As shown in the journal co-citation network visualization map (**Figure 6A**), the size of the node indicates the centrality, which is proportional to the number of citation times. There were 239 nodes and 28438 links and only journals with a minimum of 200 citations were included. The top 3 journals with the largest TLS were *Cancer Research* (17593), *Nature* (13624), and *Proc Natl Acad Sci U S A* (9388). A density visualization map of journal co-citation analysis was also provided in **Figure 6B**.

**Table 4:** The top 10 journals with the most attention to glioma stem cells.

Journal	Quantity	% of 4990	H-index	ACI
*Oncotarget*	202	4.048	45	35.45
*Plos One*	158	3.166	43	39.54
*Cancers*	132	2.645	24	14.8
*Cancer ResearchA*	123	2.465	61	148.62
*Neuro Oncology*	122	2.445	42	44.75
*International Journal of Molecular Sciences*	88	1.764	17	10.89
*Oncogene*	84	1.683	38	81.06
*Stem Cells*	80	1.603	45	80.36
*Cancer Letters*	75	1.503	34	48.4
*Scientific Reports*	73	1.463	22	21.53

ACI, average citations per item.

### Distribution of authors

**Figure 7:**
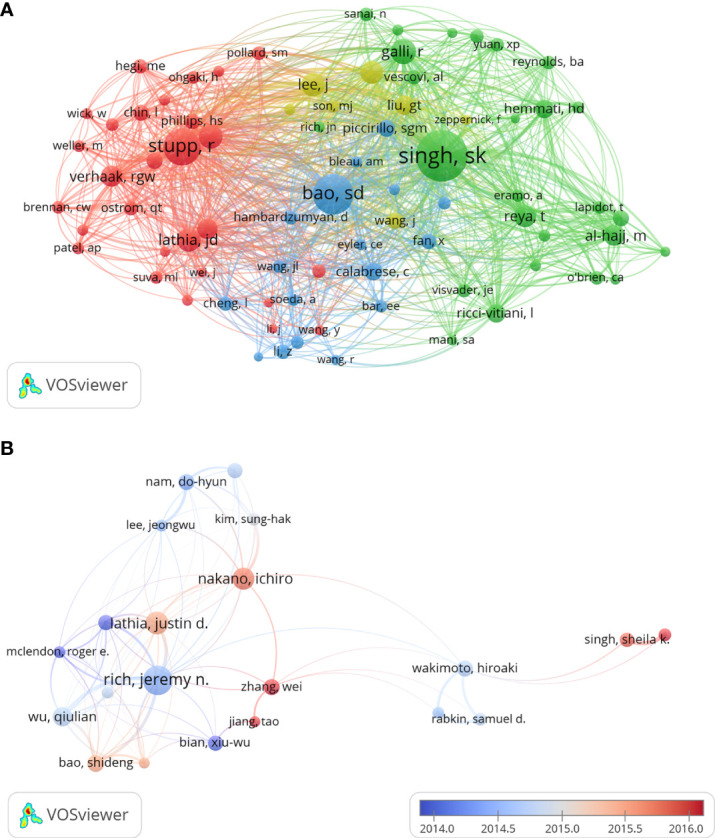
The author co-citation network map **(A)** and co-authorship overlay map **(B)** generated by VOS viewer. Each node represents an author, and node size indicates the number of publications. The purple nodes represent the author appear earlier, whereas the red nodes reflect the recent occurrence.

The top 20 productive authors published 937 papers on GSCs, accounting for 18.78% of all 4990 publications. The details of the top 20 authors published papers are shown in **Table 5**. RICH JN from the University of California, Santiago is the author with the most publications (109), comprised 2.18% of total, and the highest H-index (59), followed by LATHIA JD from Case Western Reserve University (71, 1.42%), and NAKANO I from the University of Alabama, Birmingham (67, 1.34%). Besides, of these top 20 scholars, the United States has the most authors with seven, followed by China (five authors), Korea (three authors), Italy (two authors), and Canada (two author). Notably, Dirks PB from University of Toronto is the author with the highest ACI (357.41 citations per publication), although he has published only 32 papers. The author co-citation network map in **Figure 7A** has also confirmed that SINGH SK was the author with the highest centrality (TLS=2969.37), followed by BAO SD (TLS=1995.09) and Stupp R (TLS=1782.19). As for author co-authorship analysis, a total of 37 authors with more than 20 documents were analyzed using the VOS viewer software (**Figure 7B**). The top 3 authors with the largest TLS were RICH JN, WU QL, and Bao SD. In addition, the authors of Jiang T, Zhang W and Singh Sk showed a relatively latest AAY of 2017.25, 2016.88, and 2015.83, respectively, which indicates that they are more active researchers in this field recently.

**Table 5:** The top 20 authors contributing the greatest amount to glioma stem cells.

Authors	Quantity	% of 4990	H-index	ACI	Country	Institute
Rich JN	109	2.184	59	163.37	USA	University of California San Diego
Lathia JD	71	1.423	38	91.03	USA	Case Western reserve university
Nakano I	67	1.343	33	54	USA	University of Alabama Birmingham
Wu QL	53	1.062	39	185.83	USA	Case Western reserve university
Kim SH	50	1.002	26	51.48	South Korea	Chonnam National University
Kim H	47	0.942	20	30.74	South Korea	Chonnam National University
Singh SK	46	0.922	23	237.83	Canada	McMaster University
Bao SD	41	0.822	33	200.73	USA	Cleveland Clinic Foundation
Zhang W	41	0.822	20	38.2	Peoples R China	Beijing Neurosurgical Institute
Zhang Y	41	0.822	21	41.17	Peoples R China	Shanxi Medical University
Liu Y	40	0.802	17	31.65	Peoples R China	Anhui University of Science and Technology
Hjelmeland AB	39	0.782	28	254.31	USA	University of Alabama Birmingham
Bian XW	38	0.762	28	54.24	Peoples R China	Third Military Medical University
Pallini R	38	0.762	18	64.34	Italy	Catholic University of the Sacred Heart
Ricci-vitiani L	38	0.762	19	67.76	Italy	Istituto Superiore di Sanita
Taylor MD	38	0.762	21	62.53	Canada	University of Toronto
Nam DH	37	0.741	25	59.76	South Korea	Sungkyunkwan University
Wakimoto H	37	0.741	24	58.7	USA	Harvard University
Wang Y	34	0.681	18	42.06	Peoples R China	Third Military Medical University
Dirks PB	32	0.641	24	357.41	Canada	University of Toronto

### Reference analysis

**Table 6** demonstrates the top 10 most frequently cited papers. Among them, 80% were based on original research, and 9 studies were published prior to 2010. All these studies were published in top-rank journals including 3 in *Nature*, 2 in *Cancer Research*, and so on. And all of them were co-cited more than 1300 times. The article by SINGH et al. published in 2004 was the most cited (5417 citations) paper, and the article is entitled “Identification of human brain tumor initiating cells”. At the same time, the article by Bao et al. entitled “Glioma stem cells promote radio resistance by preferential activation of the DNA damage response” and the article by SINGH et al. entitled “Identification of a cancer stem cell in human brain tumors” also received enormous attention and each of them acquired more than 3000 citations.

**Table 6:** The 10 most cited papers of research on glioma stem cells from 2003 to 2021.

Title	Journal	First author	Year	Number of institutions	Citation	Document type
Identification of human brain tumour initiating cells	*Nature*	Singh SK	2004	1	5417	Article
Glioma stem cells promote radioresistance by preferential activation of the DNA damage response	*Nature*	Bao SD	2006	1	4324	Article
Identification of a cancer stem cell in human brain tumors	*Cancer Res*	Singh SK	2003	1	3729	Article
Malignant gliomas in adults	*N Engl J Med*	Wen PY	2008	2	2960	Review
Identification of pancreatic cancer stem cells	*Cancer Res*	Li CW	2007	3	2433	Article
Malignant astrocytic glioma: genetics, biology, and paths to treatment	*Genes Dev*	Furnari FB	2007	1	1715	Review
Identification of a subpopulation of cells with cancer stem cell properties in head and neck squamous cell carcinoma	*Proc Natl Acad Sci U S A*	Prince ME	2007	3	1616	Article
A perivascular niche for brain tumor stem cells	*Cancer Cell*	Calabrese C	2007	1	1550	Article
A restricted cell population propagates glioblastoma growth after chemotherapy	*Nature*	Chen J	2012	1	1377	Article
Analysis of gene expression and chemoresistance of CDI33(+) cancer stem cells in glioblastoma	*Mol Cancer*	Liu GT	2006	1	1317	Article

### Keyword analysis

**Figure 8:**
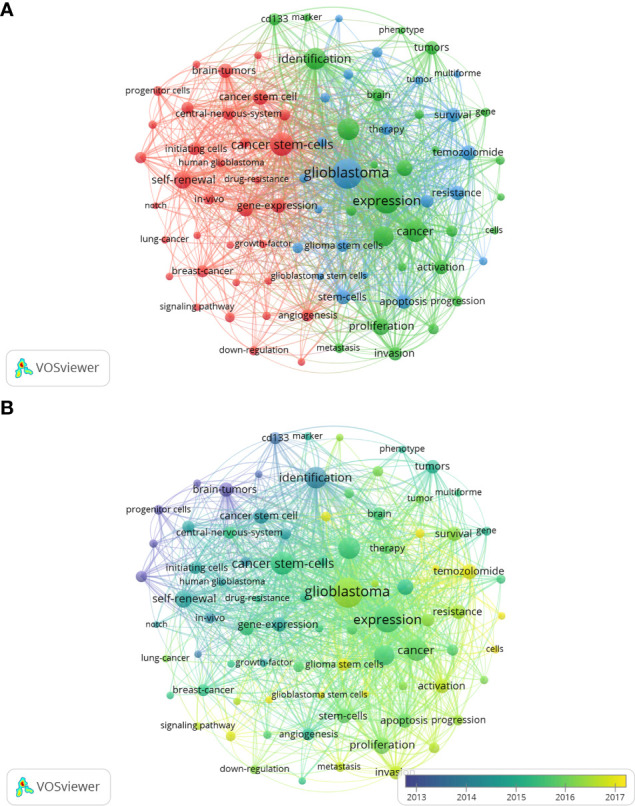
The network visualization map **(A)** and overlay visualization map **(B)** of keywords co-occurrence analysis on glioma stem cells research. Keywords with similar categories are gathered in a cluster. There are three clusters, including glioma stem cell properties” (red nodes), “cell biological properties” (green nodes) and “oncology therapy” (blue nodes).

The goal of keywords co-occurrence analysis is to trace scientific development and identify potential hot topics of a certain field. By applying VOS viewer, we extracted 41 keywords that appeared more than 200 times, of which 39remained after manually removing the duplicates. As shown in the **Figure 8A**, all the identified keywords were classified into 3 main clusters: “glioma stem cell properties” (red nodes), “cell biological properties” (green nodes) and “oncology therapy” (blue nodes). The keyword “glioblastoma” appeared most frequently, with 1690 occurrences, followed by expression (1251), caner stem cells (1014). In the cluster of “glioma stem cell characteristics”, the prominent keywords were self-renewal (565), brain tumors (403), and gene expression (377). As for the cluster of “cell biology”, expression (1278), glioma (903), identification (887) and growth (742) were the most frequently appearing words. In the third keyword cluster of “oncology therapy”, studies on temozolomide (437), survival (397), stem cell (394) and resistance (386) are common.

As shown in the keywords overlay visualization map (**Figure 8B**), the keywords “progenitor cell”, “CD133” and “identification” appeared earlier with the smaller AAY, which revealed that numbers of studies were focused on the identification and characterization of GSCs in the early stages of glioma stem cell research. While since 2016, research focus has shifted to “growth”, “proliferation”, “invasion”, “apoptosis”, that is the biological characterization of glioma. Further, the emergence of keywords “radiotherapy” and “chemotherapy”, which has the latest AAY, are seen as a shift in focus from basic cellular biology to clinical problem solving.

In order to validate this conclusion, we further analyzed the most cited papers between 2012-2016, as well as 2017-2021. As displayed in **Supplement Table 1**, the most cited articles between 2012-2016 was mainly about the topics of glioma stem cells, tumor microenvironment, and chemoresistance. While the analysis of the top 10 most cited articles from 2017 to 2021 showed that Temozolomide and epithelial-mesenchymal transition emerged as new issues for scholars and immunotherapy with glioma stem cells has also attracted great attention from researchers in this field (**Supplement Table 2**). Therefore, the above result could lead to the conclusion that cancer therapy resistance and immunotherapy with glioma stem cells will be the potential research hotspots in the near future.

## Discussion

Unlike systematic reviews, bibliometrics is a type of document analysis method that integrates mathematics, statistics, philology, and other disciplines (20, 23), and can be employed to assess the scientific output in a certain field. In this study, we chose the WOS database as the main source of data (19) and then VOS viewer software (28) was used to visualize bibliometric networks of literature information related to GSCs research from 2003 to 2021.

To the search date, a total of 4990 publications with 226043 cited times were identified, and the annual volume of publications on GSCs showed an increasing trend from 2003 to 2021. Among 85 countries participated in the publication of this domain, the United States was the most prolific country accounting for more than one third of the global total publications, followed by China and Italy. As can be seen from the annual number of publications in the top five prolific countries, the United States consistently maintains its leading position in this field during the period 2003-2021. Similarly, there has been an upward trend on the whole in annual number of publications in China and the gap with the United States gradually narrowed. This finding indicates that the studies of GSCs have attracted the interest of many Chinese scholars. It’s quite predictable that the investment to GSCs research will be further strengthened globally, especially in the United States and China.

H-index is often used to measure both the productivity and academic impact of a scholar, institution or country. Specifically, if a scientist has n articles cited more than n times, then his/her H-index is n (27, 28). Moreover, ACI and SOTC are the additional indicators that widely used to describe academic contributions (20, 25). Our results suggest that the United States had the highest H-index (157) and the second largest AIC (67.54), which indicates that the United States not only published a large number of publications, but the quality of the publications was also highly valuable and informative in academic terms. Besides, the United States accounted for 70% of the top 10 productive institutions on GSCs research. University of California System in the United States had the large number of publications (205) and the high SOTC (13741). This may be an important reason why the United States has an irreplaceable position in the field of GSCs research.

Also, although the overall number of publications and H-index from China in this field ranked the second to the United States, the AIC was only 31.33 times. Therefore, except for increasing production, China also needs to enhance the innovativeness and depth of related research. Importantly, Zhang Y in Shanxi Medical and Zhang W in University Beijing Neurosurgical Institute both have the largest quantity of publications (41), indicating they pay plenty of attention in this field. Wang Y of Third Military Medical University obtains a large number of high-quality research results with the greatest AIC of 42.06. They are more likely to be funded and change the current largely homogeneous state of affairs.

In addition, as one of the early pioneers to focus on GSCs, although Canada ranked seventh in terms of publication quantity, its ACI is the highest (85.98). The University of Toronto was the first institution to study GSCs with an ACI of 126.38, much higher than other institutions. Already in 2003, *Cancer Research* published an article by Canadian scholar Singh et al, entitled “Identification of a cancer stem cell in human brain tumors” (29). CD133, a widely known transmembrane protein expressed on the surface of hematopoietic stem cells, is the first-reported CSC marker of leukemia (30, 31). Nevertheless, this study proposed that CD133 could mark GSCs and be used for identifying and sorting GSCs, making a tremendous advance to GSCs research. Then, in 2004, Singh et al, further demonstrated in “Identification of human brain tumor initiating cells” published in *Nature* that CD133+ GSCs spheres could initiate gliomagenesis and have the potential to differentiate into normal tumor cells (32).

Co-citation analysis is a method for measuring the degree of relationship between documents. It refers to when two documents are cited by a third document at the same time, and the two documents can be considered to form a co-citation relationship. The higher the frequency of citations, the closer the academic relationship between the two documents. TLS is an important quantitative indicator to the strength of links between them and is positively correlated with the number of citations. The results of the journal co-citation analysis showed that the top three journals with the largest TLS were *Cancer Research*, *Nature*, and *Proc Natl Acad Sci U S A*. Beyond this, the top three H-index journals were *Cancer Research*, *Stem Cells* and *Oncotarget*. This indicates that some literature published in the above-mentioned journals may have significant academic impact and marvelous reference value for research in the field. Additionally, these results could also provide directions for scholars to submit related manuscripts. Moreover, authors were classified into four categories based on author co-citation analysis. The top three authors with the largest TLS were Singh Sk, BAO SD, and Stupp R. The authors of Jiang T, Zhang W and Singh Sk showed a relatively latest AAY of 2017.25, 2016.88, and 2015.83, respectively, which indicates that they are more active researchers in this field recently. However, none of these scholars have enormous cited times, which may be caused by the short time of publication.

The 10 top most frequently cited articles were distributed between 2003 and 2012. Of these 10 articles, there were 4 articles about identification of GSCs from 2003 to 2007, and 3 articles on the radiotherapy resistance of GSCs, suggesting GSCs may be considered as potential therapeutic targets in glioma. The most cited article titled “Identification of human brain tumor initiating cells” was published in *Nature* in 2004, which proposed that CD133 was a surface marker of GSCs and thus could be used for the identification and sorting of GSCs (32). Furthermore, it was demonstrated that GSCs have differentiation potential. Then, Prince ME proposed that CD44 was a surface marker in the study titled “Identification of a subpopulation of cells with cancer stem cell properties in head and neck squamous cell carcinoma” in 2007 (33). However, studies show that unlike normal stem cells, GSCs may respond to differentiation but fail to fully lose their stemness. Helena C found that GSCs-derived cells didn’t undergo terminal cell cycle arrest and remain vulnerable to de-differentiation. When differentiation stimulation being withdrawn, they may return to GSC-like cells with proliferative potential (34–36).

GSCs received a lot of attention from 2010 to 2015 as a therapeutic target for glioma because of their association with oncologic therapy resistance (37, 38) and angiogenesis (39, 40). In the last five years, the research focus was mainly on the regulation of the immune microenvironment of GSCs (41, 42), including macrophages, neutrophils, and lymphocytes. A novel approach for glioma therapy is to promote apoptosis of GSCs (5), reduce immunosuppression and suppress metastasis by regulating immune cells (43, 44).

The purpose of keyword co-occurrence analysis was to track scientific developments and to identify prospective hot topics in a domain. All the identified keywords were classified into three main clusters: “glioma stem cell properties”, ”cell biological properties” and “oncology therapy”. In the keywords overlay visualization map, keywords related to stem cells property such as “identification”, “CD133” and “marker”, occurred extremely frequently and appeared quite early, indicating early studies were mainly devoted to the identification of GSCs (29, 32, 33). Then, the keywords “apoptosis”, “proliferation” and “invasion” appeared which were about cell biological properties (45, 46). After GSCs were marked and sorted, researchers began to use tools to regulate GSCs’ growth, such as LncRNA or miRNA to alter cellular gene expression (12–14). These basic researches have provided new insights into the glioma treatment. In 2015, the terms of clinical relevance “angiogenesis” and “resistant” emerged frequently. The research task of many research teams during this period is mainly to find mechanisms of clinical symptoms on the cellular and genetic level (47). It is not hard to see that research hotspot is beginning to move from basic cellular studies to the solution of clinical problems.

Recently, keywords that appeared at a high frequency including “temozolomide” and “epithelial-mesenchymal transition” suggests that chemotherapy resistance in gliomas is currently the hot topic of GSCs research. TMZ is the first-line chemotherapy drug for glioma. A multitude of clinical studies showed that there was a significant decrease in the treatment effect in a subset of patients when TMZ treatment was repeated (13, 48). Currently, GSCs are considered as the main cause of the TMZ tolerance to glioma. TMZ prevents DNA replication by guanine methylation of DNA, causing cells to arrest in M phase. Target cells of TMZ are tumor cells with robust growth and metabolism instead of silent GSCs in quiet G0 phase (10). Therefore, most normal gliomas cells are killed and chemotherapy-insensitive GSCs are preserved following the first exposure to TMZ treatment. However, when TMZ treatment is repeated, the tumor cell killer effect of oncology therapy is strongly reduced. MGMT is mainly involved in DNA methylation repair and highly expressed in GSCs (9, 11). It is reported that there was a strong positive correlation between MGMT activity and TMZ tolerance of tumors in gliomas. It is a decent therapeutic approach to reduce TMZ resistance and improve the effect of TMZ by inhibiting MGMT activity and decreasing its expression. In addition, GSCs highly expresses the anti-apoptotic gene Bcl-2 (49) and ATP-binding cassette transporter (50) to pump drugs out of the cell and protect the tumor cell from oncology therapy. GSCs release extracellular vesicles, which is involved in therapeutic resistance. At the present time, studies are mainly aimed at promoting GSCs apoptosis and inhibiting GSCs proliferation. For example, LncRNA or miRNA promote GSCs apoptosis and autophagy (12, 13, 47). Generally, treatment combinations have a stronger effort against TMZ tolerance. Combinated treatment of photodynamic therapy or sonodynamic therapy with TMZ could kill tumor stem cells (38, 51). Recently, tumor treating fields (TTFields) is a novel tumor treatment. Paul C et al. demonstrated that TTFields with TMZ inhibited GSCs proliferation and tumor sphere formation (52). Theoretically, it is a potential treatment to promote GSC differentiating and escaping from quiescence for enhancing therapeutic sensitivity (53–55). Whereas, there are few reported clinical application about GSCs.

Epithelial-to-mesenchymal transition (EMT) is a cellular mechanism which is known to promote normal embryonic development (56, 57). EMT is a transcriptional process in which epithelial cells take on mesenchymal properties through loss of cell-to-cell adhesion, acquisition of migration and invasiveness, and loss of cell polarity (58). In general, EMT contributes to tumorigenesis, invasion, distant metastasis, and resistance to chemotherapy and/or radiotherapy in tumors. Significantly, in cancer, EMT is a dynamic process of mutual transformation of epithelial cells to mesenchymal cells. To some extent, tumor cells acquire stem cell-like properties, increased motility and invasive capacity through EMT (59). It also promotes glioma immune evasion and immunosuppression and enhances resistance to current oncology therapy. There are important pathways involved in epithelial mesenchymal transition that also maintain the stemness of GSCs such as Wnt/β-catenin pathway, SHH pathway and NOCTH pathway (60). The Wnt/β-catenin pathway combined with TGF-β signaling induces EMT through high expression of HIF-α (40). Critically, Frizzled-related protein (sFRP4), as a Wnt pathway antagonist, reverses EMT and reduces drug resistance. It is known that activation of receptor tyrosine kinases (RTK), such as epidermal growth factor and FGF, can induce EMT by activating the classical PI3K/AKT and RAS/MAPK pathways (61). Clinical trials have been conducted with STAT3 inhibitors for the treatment of cholangiocarcinoma, breast and ovarian cancers.

The neoplasm is surrounded by a multitude of non-tumor cells that constitute a complex tumor immune microenvironment, including dendritic cells, microglia, lymphocytes, tumor-associated macrophages, and endothelial cells (42, 62). GSCs recruit monocytes by secreting CSF-1 and CCL2 (63), upregulate STAT3 expression, and promote the conversion of monocytes to M2 tumor-associated macrophages, which contribute to angiogenesis and tumor metastasis (42). In addition, M2 tumor-associated macrophages can contribute to GSCs immunosuppression through the secretion of immunosuppressive factors such as IL-10 and TGF-b1 (64). Therefore, M2 tumor-associated macrophage is a potential immunotherapeutic target for glioma stem cells (65). Besides, attracted oligodendrocytes and macrophages/microglia provide advantages for GBM in the development of the GSC niche in proliferation, migration, and stemness (66). In addition, tumor-associated macrophages expressed CD204 specifically engulf GSC-derived necrotic particles, and then upregulated the expression of IL-12 which enhance the sphere-forming activity of GBM patient-derived cells (67). Therefore, tumor-associated macrophages hold great promise to eliminate glioma. Glioma cells invade along some vital anatomical structures, including the white matters. Jun W et al. demonstrated that CD133+ GSCs are more likely to locate on the jagged1+ (a Notch ligand) nerve fibers. Importantly, a Notch1-Sox9-Sox2 positive feedback loop enhance the ability of GSCs to survive in WM tracts by enhancing stemness, thereby promoting invasive growth (68). When tumor invasion into the white matters induces an injury-like microenvironment, the white matter suppresses malignancy by directing GSC differentiation towards pre-oligodendrocyte fate. But once removed from white matter, GSC-derived oligodendrocytes reverse a GSC state. Continuous exposure to white matter is a condition of GSCs differentiation (69). Brain tumor metastasis is usually accompanied with destruction of blood vessels and blood brain barrier. Endothelial cells prompt GSCs self-renewal through activating Notch and Hedgehog pathway (70, 71). Hypoxic environment prompt GSCs stemness maintenance. In peri-hypoxic niches, hypoxia-induced factors are important in the regulation of stemness (72). Hypoxic environment modification probably is perpetual topic on tumor treatment.

### Strengths and limitations

There are many systematic reviews but no bibliometric studies on GSCs. Our study is the first bibliometric study on GSCs. Bibliometric analysis combined with visualized maps can provide systematic information about GSC-related studies. There are several important advantages of our study. First, the visualized map can help readers to learn about the evolution of GSCs research and current research hotspots relatively easily. Second, keyword analysis makes it easy for researchers to capture potential research targets. Finally, co-author analysis and co-citation analysis in terms of country, institution and author could provide references for scientists and funding agencies.

However, this study still has several limitations. Because the language of the included studies was limited to English, it may have overlooked several important studies published in other languages. There are inconsistencies between the results of the bibliometric analysis and the status of the actual studies. This is due to the fact that the database on which this study is based stays open and updated. In addition, the growing trend in the number of published papers may last longer than predicted.

## Conclusions

This study summarizes the current state and global trends in GSCs research. There is an increasing trend in the number of publications and the United States is in a leading position of this domain. The Cleveland Medical Center holds the largest number of publications, citations, and the highest value of H-index. RICH JN and SINGH SK were the key authors. According to keyword co-occurrence analysis, all the identified keywords were classified into three main clusters: “glioma stem cell properties”, ”cell biological properties” and “oncology therapy”. In particular, the research focus is gradually shifting from “glioma stem cell properties” to “oncology therapy”. Based on the analysis of top cited articles, research on “immunothreapy”, “epithelial-mesenchymal transition”, and “temozolomide resistance” will be the next potential research hotspots and may create new therapeutic strategies for GSCs.

## Data availability statement

The raw data supporting the conclusions of this article will be made available by the authors, without undue reservation.

## Author contributions

In review, HY and XGT designed the study. SRS and HYW collected the data. SRS, HYW, FCW, JJJ, LXX, HGW, XGT and HY analyzed the data and drafted the manuscript. HY, HGW and XGT revised and approved the final version of the manuscript. All authors contributed to the article andapproved the submitted version.

## Funding

This research was supported by the grants awarded by Natural Science Foundation of Tianjin Municipal Science and Technology Commission (20JCQNJC00410).

## Acknowledgments

The authors thank Dr. Jiao Dian and Dr. Duan Chenyang for their help in language polishing.

## Conflict of interest

The authors declare that the research was conducted in the absence of any commercial or financial relationships that could be construed as a potential conflict of interest.

## Publisher’s note

All claims expressed in this article are solely those of the authors and do not necessarily represent those of their affiliated organizations, or those of the publisher, the editors and the reviewers. Any product that may be evaluated in this article, or claim that may be made by its manufacturer, is not guaranteed or endorsed by the publisher.

## Abbreviations

GSC, Glioma stem cell; ACI, Average citations per item; TLS, Total link strength; SOTC, Sum of the times cited; TMZ, Temozolomide; MGMT, O-6-Methylguanine-DNA methyltransferase; SCI-EXPANDED, Science Citation Index-Expanded; WoSCC, Thomson Reuters’ WoS Core Collection; WoS, Web of Science; AAY, Average appearing year; EMT, Epithelial-to-mesenchymal transition.
