# Supplementary material for: Global research trends and hotspots on glioma stem cells
Source: Front Oncol. 2022 Sep 29;12:926025. doi: 10.3389/fonc.2022.926025 (PMC9558893; doi:10.3389/fonc.2022.926025)
Supplement: Supplementary file 1 [file Table_1.docx]

**Supplement table 1 The 10 most cited papers of research on glioma stem cells from 2012 to 2016**

| Title | Journal | First author | Year | Number of institutions | Number of countries | Citation | Document type |
| --- | --- | --- | --- | --- | --- | --- | --- |
| A restricted cell population propagates glioblastoma growth after chemotherapy | *Nature* | Chen J | 2012 | 1 | 1 | 1377 | Article |
| Cancer stem cells in glioblastoma | *[Gene Dev](https://www.medsci.cn/sci/submit.do?id=3ac32549)* | Lathia JD | 2015 | 2 | 1 | 801 | Review |
| Engineered T cells: the promise and challenges of cancer immunotherapy | *Nat Rev Cancer* | Fesnak AD | 2016 | 2 | 1 | 599 | Review |
| Glioblastoma Stem Cells Generate Vascular Pericytes to Support Vessel Function and Tumor Growth | *Cell* | Cheng L | 2013 | 4 | 2 | 519 | Article |
| Single-cell RNA-seq supports a developmental hierarchy in human oligodendroglioma | *Nature* | Tirosh I | 2016 | 3 | 1 | 440 | Article |
| Periostin secreted by glioblastoma stem cells recruits M2 tumour-associated macrophages and promotes malignant growth | *[Nat Cell Biol](https://www.medsci.cn/sci/submit.do?id=5df95069)* | Zhou WC | 2015 | 3 | 1 | 440 | Article |
| CCAT2, a novel noncoding RNA mapping to 8q24, underlies metastatic progression and chromosomal instability in colon cancer | *Genome Res* | Ling H | 2013 | 15 | 8 | 439 | Article |
| Mesenchymal glioma stem cells are maintained by activated glycolytic metabolism involving aldehyde dehydrogenase 1A3 | *PNAS* | Mao P | 2013 | 5 | 3 | 384 | Article |
| Malignant Glioma: Lessons from Genomics, Mouse Models, and Stem Cells | *Cell* | Chen J | 2012 | 1 | 1 | 366 | Review |
| Glioblastoma multiforme: Pathogenesis and treatment | *Pharmacol Therapeut* | Alifieris C | 2015 | 1 | 1 | 343 | Review |
