# Supplementary material for: Global research trends and hotspots on glioma stem cells
Source: Front Oncol. 2022 Sep 29;12:926025. doi: 10.3389/fonc.2022.926025 (PMC9558893; doi:10.3389/fonc.2022.926025)
Supplement: Supplementary file 2 [file Table_2.docx]

**Supplement table 2 The 10 most cited papers of research on glioma stem cells from 2017 to 2021**

| Title | Journal | First author | Year | Number of institutions | Number of countries | Citation | Document type |
| --- | --- | --- | --- | --- | --- | --- | --- |
| The Microenvironmental Landscape of Brain Tumors | *Cancer Cell* | Quail DF | 2017 | 2 | 2 | 594 | Article |
| m(6)A RNA Methylation Regulates the Self-Renewal and Tumorigenesis of Glioblastoma Stem Cells | *Cell Rep* | Cui Q | 2017 | 4 | 2 | 581 | Article |
| Current state of immunotherapy for glioblastoma | *Nat Rev Clin Oncol* | Lim M | 2018 | 2 | 2 | 416 | Review |
| Every step of the way: integrins in cancer progression and metastasis | *[Nat Rev Cancer](https://www.medsci.cn/sci/submit.do?id=87107084)* | Hamidi H | 2018 | 2 | 1 | 288 | Review |
| Packaging and transfer of mitochondrial DNA via exosomes regulate escape from dormancy in hormonal therapy-resistant breast cancer | *PNAS* | Sansone P | 2017 | 7 | 4 | 281 | Article |
| Intra-tumor heterogeneity from a cancer stem cell perspective | *[Mol C](https://www.medsci.cn/sci/submit.do?id=b4d34816)ancer* | Prasetyanti PR | 2017 | 4 | 1 | 251 | Review |
| Macrophage Polarization Contributes to Glioblastoma Eradication by Combination Immunovirotherapy and Immune Checkpoint Blockade | *Cancer Cell* | Saha D | 2017 | 3 | 1 | 250 | Article |
| Coding and noncoding landscape of extracellular RNA released by human glioma stem cells | *[Nat Commun](https://www.medsci.cn/sci/submit.do?id=a48311372)* | Wei ZY | 2017 | 10 | 4 | 225 | Article |
| Tumor-derived spheroids: Relevance to cancer stem cells and clinical applications | *[Cancer Sci](https://www.medsci.cn/sci/submit.do?id=ce902925)* | Ishiguro T | 2017 | 2 | 1 | 216 | Review |
| Overcoming therapeutic resistance in glioblastoma: the way forward | *[J Clin Invest](https://www.medsci.cn/sci/submit.do?id=753b3596)* | Osuka S | 2017 | 1 | 1 | 206 | Review |
